# A novel index to measure pre‐planning in the Tower of London task: Test–retest reliability and known‐group validity

**DOI:** 10.1111/bjop.70044

**Published:** 2025-11-28

**Authors:** Lena V. Schumacher, Benjamin Rahm, Christoph P. Kaller, Valentin Schyle, Cornelius Weiller, Josef M. Unterrainer

**Affiliations:** ^1^ Institute of Medical Psychology and Medical Sociology, Faculty of Medicine University of Freiburg Freiburg Germany; ^2^ Department of Neuroradiology, Medical Center – University of Freiburg, Faculty of Medicine University of Freiburg Freiburg Germany; ^3^ Department of Neurology and Neuroscience, Medical Center – University of Freiburg, Faculty of Medicine University of Freiburg Freiburg Germany

**Keywords:** intra‐class correlation coefficients, known‐group validity, neurological patients, planning, test–retest reliability, Tower of London

## Abstract

The Tower of London (TOL) is a planning task frequently used in clinical settings and research. Planning and execution times are the most common outcome variables despite yielding lower effect sizes in clinical group comparisons and lower test–retest reliability than planning accuracy. Here, it is proposed that planning time be analysed not in isolation, but in relation to the combined duration of planning and execution, yielding a novel pre‐planning index (PPI). In *N* = 179 healthy participants, test–retest reliability analyses yielded higher absolute agreement and less intra‐individual variability over two sessions for PPI than for planning and execution times. The clinical validity of PPI was probed by comparing patients known to exhibit planning deficits and healthy controls. Stroke and Parkinson's patients showed significantly lower PPI than controls, driven by reduced planning and longer execution times. There was no difference in PPI between patients with mild cognitive impairment and controls. Consistently across healthy participants and patients, the positive correlation of PPI with planning accuracy exceeded that of planning times with accuracy. Thus, this pre‐planning index can enhance both the reliability and clinical validity of TOL latency variables and represents a useful complement to accuracy for measuring planning performance in health and disease.

## BACKGROUND

Executive functions comprise higher order cognitive processes enabling conscious behavioural regulation in response to external demands or internal goals (Diamond, 2013; Norman & Shallice, 1986). They become essential when established action routines are insufficient for situational requirements or unavailable, as is the case in novel and complex situations (Burgess, 1997; Diamond, 2013; Shallice & Burgess, 1991). Dysexecutive symptoms – caused by brain lesions, neuropsychiatric disorders, ageing or other conditions – disrupt adaptive behaviour, impairing the ability to initiate, monitor and complete goal‐directed actions. Consequently, impaired executive functions undermine daily functioning and autonomy (for a review, see Diamond, 2013), highlighting their critical role in cognitive performance.

In theoretical accounts, planning is regarded as a prototypical executive function (Burgess, 1997; Norman & Shallice, 1986; Shallice & Burgess, 1991). Planning in terms of a mental ‘look‐ahead’ process is needed whenever a goal is not directly accessible, so that the steps required to reach the desired outcome must be identified while mentally anticipating and evaluating the potential consequences of goal‐directed actions before executing them (Morris & Ward, 2005). Thus, planning directly taps into the capacity for top‐down modulated cognitive processing that epitomizes executive functioning. In a wider sense, planning exemplifies the unique capacity of the human brain to transform external stimuli into internal mental representations that can then be manipulated independent of sensory input or overt actions and can be integrated into hierarchical mental structures that give rise to abstract thought across various cognitive domains (Weiller et al., 2022, 2025).

The assessment of planning abilities has commonly relied on disc‐transfer tasks such as the Tower of Hanoi (TOH) and the Tower of London (TOL; for an overview, see Berg & Byrd, 2002). Disc‐transfer tasks demand that a given start state of balls or discs distributed on pegs be transferred into a predefined goal state by moving the balls/discs from one peg to another, usually within the minimum number of moves possible (cf. Shallice, 1982). They feature well‐defined problems of varying complexity that cannot be solved by prior knowledge as well as a limited range of eligible operators for transforming the problem states. Thus, disc‐transfer tasks allow for an adequate and comparable operationalization of performance – both in terms of accuracy and latency – across individuals and have become the standard approach for measuring planning abilities (Berg & Byrd, 2002; Kaller et al., 2016; Kaller, Unterrainer, & Stahl, 2012; Sullivan et al., 2009).

Classically, performance on disc‐transfer tasks has been measured by the percentage of problems solved in the minimum number of moves possible, that is, by solution accuracy (Borys et al., 1982; Klahr, 1985; Shallice, 1982; Spitz et al., 1982). Solution accuracy is not only a conceptually straightforward operationalization of planning performance, it is also the outcome variable that yields the highest effect size in comparisons between various neurological or psychiatric diseases and healthy participants (mean Cohen's *d* = 1.43; *n* = 24 comparisons), as reported in a meta‐analysis on disc‐transfer tasks by Sullivan et al. (2009). However, there is a preponderance of measuring planning performance by latency variables in studies on disc‐transfer tasks. The initial thinking time (ITT) – the time from problem presentation until the first ball/disc is moved, indicating the duration of planning – is the most commonly used outcome measure (*n* = 37 comparisons) despite yielding lower effect sizes than accuracy (mean Cohen's *d* = 1.02) (Sullivan et al., 2009). The movement execution time (MET) – the time from the start of the first move until problem completion – is also slightly more often utilized (*n* = 26 comparisons) than the percentage of solutions in the minimum number of moves (Sullivan et al., 2009).

Given the ubiquitous utilization particularly of the TOL for assessing planning ability in healthy participants as well as in neurological or psychiatric diseases such as traumatic brain injury, neurodegenerative disorders, schizophrenia and others (for overviews, see Berg & Byrd, 2002; Sullivan et al., 2009), it is highly relevant to establish adequate reliability of the task. This is however challenging particularly in terms of test–retest reliability, because assessment of executive functions relies on task novelty to induce effortful, non‐routine cognitive processing which is difficult to maintain on repeated testing (Burgess, 1997; Phillips, 1997). For the TOL, test–retest reliability of the percentage of problems solved in the minimum number of moves and related measures of planning accuracy has been studied numerously, with the majority of studies indicating moderate (Lowe & Rabbitt, 1998; Schyle et al., 2023; Tunstall et al., 2014) or adequate reliability (Köstering, Nitschke, et al., 2015; Schnirman et al., 1998; Welsh et al., 2000) and some studies showing low reliability (Lemay et al., 2004; Syväoja et al., 2015).

By contrast, evidence on the test–retest reliability of latency variables for the TOL is surprisingly sparse. In a sample of older adults tested on three occasions, planning times yielded good reliability with indices above .80 (Lemay et al., 2004). In young adults however, only low test–retest reliability has been found for both planning and execution times on the TOL, with indices not exceeding .50 (Köstering, Nitschke, et al., 2015; Tyburski et al., 2021). Furthermore, sample sizes in all three studies were below *N* = 30, limiting the conclusions that can be drawn from these results. One of the reasons for the limited test–retest reliability particularly in young adults could be that latency measures are particularly affected by greater task familiarity on repeated testing. In the study by Köstering, Nitschke, et al. (2015), overall there was a marked decrease in ITT and MET and a moderate increase in accuracy from first to second session, but there was also a trend for a *positive* correlation of the difference scores (Session 2 minus Session 1) for accuracy and ITT. That is, participants who increased their accuracy from first to second testing session also had increased or rather *less decreased* ITT on the second session (Köstering, Nitschke, et al., 2015). That is, presumably driven by unspecific effects of task familiarity rather than by improved planning accuracy, participants seem to become faster overall on repeated testing, which considerably compromises the test–retest reliability of latency variables on the TOL.

As an approach to overcome this problem, it is here proposed that ITT – the most frequently used latency variable (Sullivan et al., 2009) – is not analysed directly, but relative to the sum of ITT plus MET, thereby yielding a ratio of ITT to overall solution time. Mathematically speaking, if ITT and MET are both markedly decreased on repeated testing (cf. Köstering, Nitschke, et al., 2015), this should affect the proposed ratio of ITT to ITT plus MET to a much lesser extent given that all three terms of the equation are reduced in parallel. That is, the proposed ratio should be more robust against general reductions in latency than ITT and MET on their own. Likewise, inter‐individual differences in general psychomotor speed that affect both ITT and MET are cancelled out in this ratio, which is particularly relevant when comparing clinical groups with control participants.

Conceptually speaking, this ITT to ITT plus MET ratio could provide more information than ITT alone as it measures how much of the overall time needed for trial completion a participant devotes to the planning phase and can thus be regarded as an index of meta‐cognitive planning (Li et al., 2015). For tasks that demand planning a solution, it is proposed that relatively more time invested in pre‐planning a solution should result in increased accuracy (Li et al., 2015, 2020). For the Sokoban puzzle – a visuo‐spatial task that demands planning ahead moves prior to their execution similar to the TOL – it was indeed shown that the pre‐planning ratio accounted for more variance in accuracy scores than planning time alone; furthermore, this ratio was significantly positively associated with the number of solutions not only on the Sokoban task itself, but also with accuracy on the TOL (Li et al., 2015). For the TOL task itself, participants instructed to fully pre‐plan their moves have higher accuracy than those instructed to immediately start solving problems (Unterrainer et al., 2003) and good performers increase their ITT from easy to more complex problems to a greater degree than low performers (Unterrainer et al., 2004). That is, a positive association between ITT and accuracy on the TOL is evident, but a pre‐planning ratio and its association with accuracy have not been investigated so far.

In sum, despite the preponderance of measuring performance on the TOL and related disc‐transfer tasks by latency variables, due to their susceptibility to changes in general speed it is questionable whether they possess adequate reliability particularly in terms of test–retest reliability and sufficient validity for revealing significant differences in clinical comparisons. The major aim of the current study was thus twofold: First, the test–retest reliability of latency measures was investigated in a large sample of young adults and it was hypothesized that computing the ratio of ITT to ITT plus MET – henceforth referred to as the pre‐planning index (PPI) – yields higher reliability than the separate latency measures. Second, the known‐group validity as a facet of construct validity (de Vet et al., 2011) was probed by testing whether PPI yields significant differences between healthy participants and three neurological populations known to exhibit planning deficits, namely patients after ischaemic stroke, with Parkinson's syndromes and mild cognitive impairment. Pre‐planning in clinical populations has not been investigated so far, but could yield valuable information on the nature of planning deficits (e.g. whether patients with lower planning accuracy also exhibit less pre‐planning). It was hypothesized that differences between clinical groups and healthy controls for PPI can be found and are at least comparable in effect size to those of the separate latency measures. Furthermore, as a third aim, it was tested whether PPI and accuracy are positively associated, which would further attest to the validity of the pre‐planning index as an outcome measure of planning performance.

## MATERIALS AND METHODS

### Participants

Reliability analyses were based on individual participant data from a previous study on test–retest reliability of the Tower of London–Freiburg (TOL‐F; Schyle et al., 2023). Participants in that study were young, healthy volunteers without a history of neurological/psychiatric diagnoses and normal or corrected‐to‐normal vision. All participants gave written informed consent prior to participation and the local ethics committee approved the study beforehand. Participants were excluded with a score above 13 on the Beck Depression Inventory (BDI‐II; Beck et al., 1996; for a full recruitment and sample description, see Schyle et al., 2023). Two consecutive sub‐samples were administered parallel or identical versions of the TOL–F, respectively, on two separate testing sessions within a 1‐week interval (Schyle et al., 2023). As demographic characteristics of the sub‐samples were very similar and test–retest reliability indices for planning accuracy were not significantly different between the sub‐samples (Schyle et al., 2023), the present analyses were carried out for the overall sample of *N* = 179 participants (mean age 21.97 years [range, 18.08–26.42 years], *n* = 97 females).

Clinical validity analyses were based on individual patient and participant data from a previous study using the TOL–F in neurological patients (Köstering, Schmidt, et al., 2015). Köstering, Schmidt, et al. (2015) recruited *N* = 60 stroke patients in the chronic stage about 6 months after ischaemic stroke, *N* = 51 patients with Parkinson's syndromes (PS; idiopathic Parkinson's disease with and without additional deep brain stimulation as well as atypical Parkinson syndromes), and *N* = 29 patients with amnestic or non‐amnestic mild cognitive impairment (MCI) as well as a larger pool of *N* = 155 healthy controls (HC) recruited from community‐dwelling citizens. All patients and HC had normal or corrected‐to‐normal vision. HC were only included without present/history of neurological or major psychiatric illnesses and without use of psychotropic medication. HC were screened for depressive symptoms with the BDI‐II (cut‐off 19 points^1^) and for signs of cognitive decline with the Montreal Cognitive Assessment (MoCA; Nasreddine et al., 2005; cut‐off 26 points). For a full description of the demographic characteristics of patients and healthy controls, and clinical information of patient groups, please refer to Köstering, Schmidt, et al. (2015). Present analyses on planning latencies are restricted to the matched groups from Köstering, Schmidt, et al. (2015), where every stroke, PS, and MCI patient was matched with a participant from the HC pool on age, sex, and education levels concurrently. Please note that from hereon, where patients and HC are directly compared, they are referred to as *groups*, whereas *sample* refers to a given patient group plus their group of matched controls. In the stroke sample (*N* = 60 patient–HC pairs), patients' mean age was 65.21 years (range, 35.25–85.50 years; *n* = 23 females^2^) and the mean age of HC was 66.25 years (43.83–81.92 years; *n* = 28 females). In the PS sample (*N* = 51 patient–HC pairs), the mean age was 64.96 years (46.25–80.50 years; *n* = 20 females) for patients and 65.81 years (43.83–82.67 years; *n* = 22 females) for HC. In the MCI sample (*N* = 29 pairs), the mean age was 74.14 years (60.67–82.25; *n* = 14 females) for patients and 73.53 years (62.83–83.33 years; *n* = 14 females) for HC.

### Tower of London–Freiburg (TOL–F)

For the two data sets analysed here, a fully computerized TOL version was used, the Tower of London–Freiburg (TOL–F; Kaller, Unterrainer, Kaiser, et al., 2012). The TOL–F uses a theory‐based task set that systematically varies three important parameters of task difficulty: the minimum number of moves to solution, the search depth and goal hierarchy of problems (for an extensive discussion of the rationale for using this structurally balanced task set, see Kaller et al., 2011; Kaller, Unterrainer, & Stahl, 2012). This yields a monotonic increase in difficulty over the minimum number of moves and good psychometric properties particularly in terms of split‐half reliability and internal consistency (Kaller et al., 2016; Kaller, Unterrainer, & Stahl, 2012). This structurally balanced task set is available in three iso‐forms, which feature identical problems, but different permutations of ball colours, so that problems look – at least superficially – different.

The TOL–F comprises three coloured balls and three rods of unequal length that can hold three, two and one ball, respectively (cf. Shallice, 1982). Participants are presented with a start configuration of the balls on the rods (in the bottom half of the screen) and a goal configuration (in the upper half of the screen) and – as in the original version (Shallice, 1982) – are instructed to transform the start into the goal state in the minimal number of moves possible within a time limit of one minute. Instructions inform about the minimum number of moves and emphasise the need to plan the solution before starting to move any balls so as to encourage initial planning (cf. Unterrainer et al., 2003). The minimum number of moves is presented on screen for the whole duration of each trial so that participants are aware of it throughout. For all data analysed here, a touchscreen administration was used to avoid performance differences due to differing familiarity in handling a computer mouse, particularly for older participants and patients.

Trials are counted as correct if participants solve a problem within the minimum number of moves within the time limit. If participants solve the problem in more than the minimum number of moves, the problem is counted as completed, but an incorrect trial. If participants do not transform the start into the goal state within the time limit, the trial is counted as a time‐out trial. The task is automatically aborted after three consecutive time‐out trials. For the TOL–F, *accuracy* refers to the sum of correct trials and can range from 0 to 24 (or from 0% to 100% if expressed as a percent), as there are 24 problems in total: eight problems each with four, five and six minimum moves. Further, the TOL–F automatically measures the *initial thinking time* (ITT; time from the presentation of a trial until the selection of the first ball to be moved) and the *movement execution time* (MET; time from the selection of the first ball to be moved until completion of the trial). Planning latencies are analysed *for all trials completed within the time limit* (i.e. irrespective of whether they were completed in the minimum number of moves or with excessive moves) or for *correct trials* only.

### Statistical analyses

#### Outcome measures

For both reliability and validity analyses, the main outcome measure is the newly proposed *pre‐planning index* (PPI). Initial thinking times (ITT) and movement execution times (MET) were analysed as secondary outcome measures. The PPI is computed as ITT/(ITT + MET) × 100 and denotes – in percent – how much of the time needed to complete a trial was spent on pre‐planning a solution. PPI, ITT and MET were all computed trial‐wise and then mean‐aggregated over all trials available for each participant/patient and each testing session (in the case of the reliability sample; also see below). To maximize the number of trials entering analyses, particularly for the patient groups, all three outcome measures were based on *all trials completed in the time limit* rather than correct trials only.

#### Reliability analyses

Present test–retest reliability analyses used the data set from Schyle et al. (2023). Whereas the analyses in Schyle et al. (2023) were restricted to planning accuracy, here the latency data were analysed. For every participant, data from two separate testing sessions were available. First, repeated‐measures analyses of variance (RM‐ANOVAs) with *session* as a within‐subject factor (Session 1 and Session 2) were computed. Second, as a direct measure of test–retest reliability, the *intra‐class correlation coefficient* (ICC) for absolute agreement – termed ICC(A,1) – was computed based on two‐way random effects models and the ICC for relative consistency – termed ICC(C,1) – was computed based on two‐way fixed effects models (McGraw & Wong, 1996). Whereas the ICC(A,1) takes the absolute difference in an individual's score over repeated testing into account, the ICC(C,1) for relative consistency only analyses the relative stability of the rank order of values over repeated testing (McGraw & Wong, 1996; Shrout & Fleiss, 1979; Weir, 2005). As the TOL–F is designed to be used in clinical settings, where intra‐individual stability in scores is of much higher importance than the rank order stability within a sample, the ICC(A,1) is reported as the main outcome measure of test–retest reliability and the ICC(C,1) is only reported for the sake of completeness and to facilitate comparisons across studies. Third, the *coefficient of variation* (CV) and the *minimal difference* (MD) are reported as measures directly quantifying the individual variation in scores over time (Atkinson & Nevill, 1998; Hopkins, 2000; Weir, 2005). The CV expresses the standard deviation of a variable as a ratio to the mean and is given in percent which makes it easily comparable across different outcome measures (Atkinson & Nevill, 1998). There are several ways of computing the CV for repeated measurements. Here a recently proposed method was used specifically suited when systematic differences between the two measurements exist (Åsberg & Bolann, 2023), as is the case for expected familiarity/practice effects in the TOL–F. In detail, the difference in percentage of the mean (DPM) was calculated for each participant, that is, individual score of Session 2 minus Session 1 divided by the individual mean over the two sessions (Åsberg & Bolann, 2023). The standard deviation of these individual DPMs was calculated and divided by √2, yielding the CV in percent (Åsberg & Bolann, 2023). The MD is calculated as the standard deviation of individual difference scores × 1.96 (Weir, 2005) and quantifies the minimally needed value that can be considered to reflect a real change in scores beyond measurement error with 95% confidence (Weir, 2005).

RM‐ANOVAs and reliability indices were computed for all three outcome measures (PPI, ITT, MET), which were mean‐aggregated for each participant and each session over all trials completed (mean [Min–Max] number of trials completed was 22.18 [10–24] for Session 1 and 23.07 [9–24] for Session 2). Furthermore, difference scores for accuracy, PPI, ITT and MET (scores of Session 2 minus Session 1; see Table 1) were correlated with each other using Kendall's tau‐b correlations. All analyses were performed with the RStudio software (version 2024.9.0.375; Posit team, 2024) running on R (version 4.4.1; R Core Team, 2024). Data preprocessing was carried out with the data.table package (version 1.16.0; Barrett et al., 2024), RM‐ANOVAs based on Type III sums of squares were computed with the rstatix package (version 0.7.2; Kassambara, 2023); ICCs and MDs were computed using the package SimplyAgree (version 0.2.0; Caldwell, 2022). CVs with 95% confidence intervals derived from the chi‐squared distribution were computed manually in R using the formula from Åsberg and Bolann (2023).

**TABLE 1:** Descriptive statistics in the reliability sample.

	Session 1			Session 2			Difference score (session 2 – session 1)		
	*M*	*SD*	Min–max	*M*	*SD*	Min–max	*M*	*SD*	Min–max
PPI (%)	60.71	10.63	21.16–82.32	61.35	9.90	28.73–79.66	0.64	6.69	−22.06 to 20.23
ITT (s)	14.23	4.57	2.83–29.09	12.25	4.24	2.62–26.02	−1.97	3.23	−10.92 to 8.46
MET (s)	8.69	2.75	3.29–19.59	7.00	1.97	3.65–12.48	−1.70	1.97	−8.31 to 5.13

Abbreviations: ITT, initial thinking time; MET, movement execution time; PPI, pre‐planning index.

#### Validity analyses

Analyses on the validity of TOL–F latency measures were based on the matched samples from Köstering, Schmidt, et al. (2015). Whereas the analyses in Köstering, Schmidt, et al. (2015) were restricted to planning accuracy, here the latency data were analysed. The known‐group validity as a form of construct validity was assessed which probes whether a test or measure is able to distinguish between groups known to differ in the construct of interest (de Vet et al., 2011; Hattie & Cooksey, 1984; Sireci & Sukin, 2013). Here, it was assessed if PPI as the main outcome measure can differentiate between patient groups known to exhibit planning deficits and healthy controls. To this aim, an ANOVA on PPI values with *group* (patients vs. matched healthy controls) as a between‐subjects factor and the matching variables *age* (as a continuous variable in years), *sex* (female vs. male) and *education level* (ranging from Level 1, primary school completed to Level 5, higher education completed) as covariates was computed for every matched sample (stroke, PS and MCI). Corresponding ANOVAs were also computed for ITT and MET. Each outcome variable was mean‐aggregated over all trials completed for each patient/participant (mean [min–max] number of trials completed was 18.27 [5–24]). Furthermore, Kendall's tau‐b correlations of accuracy with PPI, ITT and MET were calculated for each sample to probe the association of the three latency measures with accuracy for which significant differences between patients and healthy controls have already been established (Köstering, Schmidt, et al., 2015). As in the reliability sample, data pre‐processing and analysis were carried out in R with the packages *data.table* (Barrett et al., 2024) and *rstatix* (Kassambara, 2023).

## RESULTS

### Test–retest reliability

#### Descriptive statistics

**FIGURE 1:**
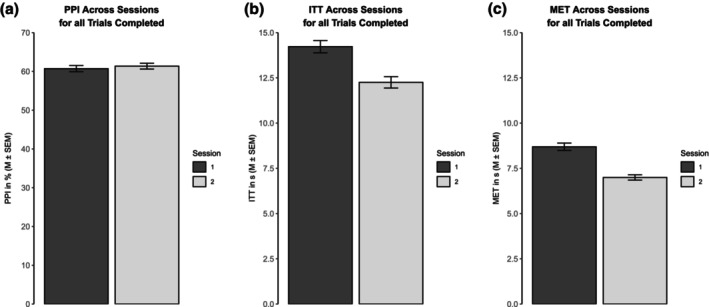
Planning latency variables across sessions in the reliability sample. Mean (a) pre‐planning index (PPI), (b) initial thinking times (ITT) and (c) movement execution times (MET) across repeated measurements (dark grey bars, Session 1; light grey bars, Session 2) in the reliability sample. Error bars denote the standard error of the mean (SEM).

For an overview on descriptive statistics, see Table 1 and Figure 1. For supplementary descriptive statistics for correct trials only, see Supporting Information, Subsection 1.1.

As is often the case for reaction time data, session‐specific PPI and MET variables were not normally distributed (Shapiro–Wilk tests: highest *W* = 0.961, all *p* < .001; for an illustration, see quantile–quantile plots in Figure S2). Hence, whereas descriptive statistics given in Table 1 and depicted in Figure 1 refer to untransformed data for ease of understanding, PPI and MET variables were transformed for the RM‐ANOVAs and ICC calculations to avoid bias in inferential statistics. For details on the transformations, please see Supporting Information, Sub‐section 1.2. ITT variables for both sessions did not significantly deviate from normality (ITT session 1: *W* = 0.994, *p* = .734; ITT session 2: *W* = 0.990, *p* = .242; also see Figure S2) and were left untransformed.

#### RM‐ANOVAs

For all RM‐ANOVAs, a generalized *η* ^2^ ($\eta_{G}^{2}$) of at least .02 corresponded to a small effect size, a $\eta_{G}^{2}$ of at least .13 to a medium effect and $\eta_{G}^{2}$ of .26 or higher to a large effect (Bakeman, 2005). The RM‐ANOVA on PPI yielded an effect of *session* (*F*_(1,178)_ = 41.943; *p* < .001) with a small effect size ($\eta_{G}^{2}$ = .027), corresponding to a small, but significant increase in PPI values (Figure 1a). The RM‐ANOVA on ITT also yielded a significant *session* effect (*F*_(1,178)_ = 66.863; *p* < .001) of small effect size ($\eta_{G}^{2}$ = .048) with a decrease in ITT from the first to the second testing session (Figure 1b). Likewise, for MET there was a significant effect of *session* (*F*_(1,178)_ = 263.644; *p* < .001) with a medium effect size ($\eta_{G}^{2}$ = .197) based on a decrease in MET from the first to the second session (Figure 1c).

#### Reliability indices

For an overview on reliability indices, see Table 2. In terms of absolute agreement, the ICC(A,1) was .7264 for PPI, .6660 for ITT and .4493 for MET. Similarly, relative consistency yielded the highest value for PPI with an ICC(C,1) of .7654. For ITT and MET, ICC(C,1) was .7317 and .6681, respectively. In terms of absolute measures of individual variability over repeated measurement, PPI yielded a coefficient of variation (CV) of 8.83% and a minimal difference (MD) of 13.13% that can be interpreted as reflecting a true change in scores (irrespective of whether this would be a negative or positive difference; Weir, 2005). The MD of 13.13% amounted to 21% of the grand mean over the two sessions (which was 61.03%). ITT yielded a CV of 17.16% and an MD of 6.36 s that is needed to reflect a true change in scores, with this MD corresponding to 48% of the grand mean (13.24 s). For MET, the CV was 16.33% and the MD was 3.87 s, corresponding to 49% of the grand mean (7.84 s). In sum, the highest reliability indices and lowest individual variability over repeated measurements were found for PPI.

**TABLE 2:** Reliability indices.

	ICC(A,1)		ICC(C,1)		CV		MD
	ICC	95% CI	ICC	95% CI	CV (%)	95% CI	
PPI	.7264	.5941; .8082	.7654	.7092; .8120	8.834	8.004; 9.858	13.1252%
ITT	.6660	.4551; .7811	.7317	.6689; .7841	17.160	15.547; 19.148	6.3262 s
MET	.4493	−.0035; .6878	.6681	.5940; .7311	16.334	14.799; 18.227	3.8695 s

Abbreviations: 95% CI, 95% confidence interval; CV, coefficient of variation; ICC, intraclass correlation coefficient; ITT, initial thinking time; MD, minimal difference; MET, movement execution time; PPI, pre‐planning index.

#### Correlations

Correlations of the difference variables (Session 2–Session 1) were significant throughout. The difference in PPI (PPI‐Diff) correlated positively with the difference in accuracy (ACC‐Diff) at *τ* = .299 (95% CI [0.159, 0.427]; *p* < .001). That is, participants who solved more problems on the second compared to the first testing session also relatively increased their pre‐planning index from first to second session. ACC‐Diff also correlated positively with ITT‐Diff (*τ* = .184; 95% CI [0.039, 0.322]; *p* < .001) and negatively with MET‐Diff (*τ* = −.269; 95% CI [−0.400, −0.127]; *p* < .001). ITT‐Diff and MET‐Diff correlated negatively (*τ* = −.112; 95% CI [−0.254, 0.035]; *p* = .026). As can be expected, PPI‐Diff also correlated with ITT‐Diff (*τ* = .484; 95% CI [0.363, 0.589]; *p* < .001) and MET‐Diff (*τ* = −.485; 95% CI [−0.590, −0.365]; *p* < .001).

In terms of session‐wise correlations, PPI was significantly associated with accuracy in a positive direction in both sessions (Session 1, *τ* = .360, Session 2, *τ* = .345). For an overview on session‐wise correlations, see Supporting Information, Subsection 1.3.

### Known‐group validity

#### Descriptive statistics

**FIGURE 2:**
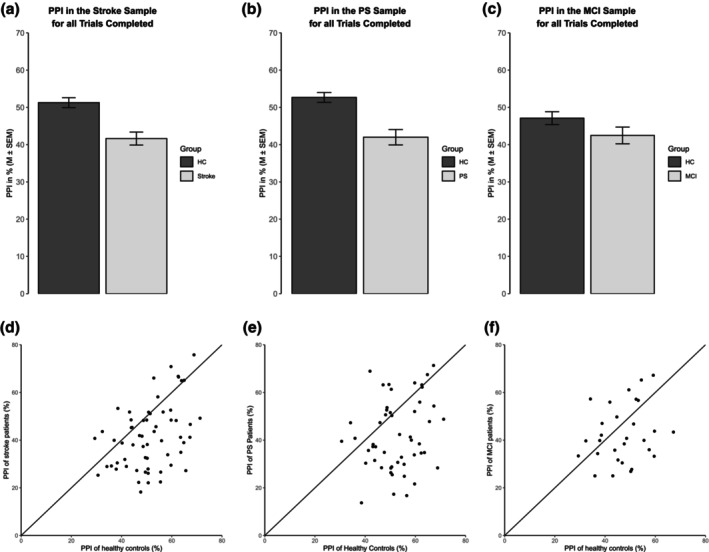
Pre‐planning index (PPI) in clinical samples. Pre‐planning index (PPI) of patients and matched healthy controls (HC) in the (a, d) stroke sample, (b, e) Parkinson syndrome (PS) sample and (c, f) mild cognitive impairment (MCI) sample. Panels a to c depict mean group‐wise PPI values (dark grey bars, HC; light grey bars, patients), with error bars denoting the standard error of the mean (SEM). Panels d to f depict pairwise scatterplots, with each data point representing one patient–HC pair. Data points above the diagonal refer to pairs where the patient has a higher PPI value than their matched HC, whereas data points below the diagonal indicate a higher PPI value in the matched HC than the patient.

For ease of interpretability, descriptive statistics refer to untransformed data, whereas variables were transformed to adhere to normality assumptions for the ANOVAs if necessary (see subsection *ANOVAs* below and Supporting Information, Subsection 2.1). See Table 3 for detailed information on descriptive statistics in the three clinical samples and Figure 2, panels a–c, for a graphical illustration.

**TABLE 3:** Descriptive statistics in clinical samples.

	Group	Stroke			PS			MCI		
		*M*	*SD*	Min–max	*M*	*SD*	Min–max	*M*	*SD*	Min–max
ACC (%)	Patients	48.82	16.00	12.50–83.33	46.81	14.99	20.83–79.17	44.54	16.56	8.33–70.83
	HC	58.26	13.10	16.67–83.33	59.48	10.84	29.17–83.33	54.89	14.22	16.67–79.17
PPI (%)	Patients	41.63	13.56	18.25–75.78	41.99	14.75	13.71–71.33	42.47	12.07	25.02–67.21
	HC	51.27	10.31	29.35–71.26	52.68	9.48	30.62–71.26	47.11	9.32	29.35–67.23
ITT (s)	Patients	10.08	5.18	3.21–25.78	9.79	5.60	2.34–20.12	10.70	5.33	3.50–24.62
	HC	12.08	3.97	4.76–20.17	12.41	3.95	4.93–20.17	11.23	4.05	4.76–19.79
MET (s)	Patients	14.60	4.13	6.43–23.98	15.17	4.29	7.74–31.90	14.41	3.64	9.24–23.84
	HC	11.82	3.62	4.62–23.23	11.48	3.09	4.62–18.19	12.93	3.54	7.34–23.23

Abbreviations: ACC, accuracy; HC, healthy controls; ITT, initial thinking time; MCI, mild cognitive impairment; MET, movement execution time; PPI, pre‐planning index; PS, Parkinson syndrome.

In addition to testing for overall group differences in PPI values (i.e. patient group vs. HC group; see below), the matched samples additionally allowed for a direct comparison of PPI values at the individual level, that is, between each patient and their matched HC. Panels d to f in Figure 2 depict pairwise scatterplots where each data point represents PPI values of a patient–HC pair, with patients' PPI values on the *y*‐axis and HC's PPI values on the *x*‐axis. That is, the points lying below the diagonal indicate those pairs where the patient had a lower PPI value than their matched HC. In the stroke sample, the patient had a lower PPI value than the respective matched HC in 71.67% of pairs (Figure 2d), with a mean (±*SD*) pairwise difference (HC–patient) of 9.63% (±13.33). In the PS sample, in 68.63% of pairs the patient had a lower PPI value than the matched HC (Figure 2e), with a mean (±*SD*) pairwise difference (HC–patient) of 10.69% (±15.82). In the MCI sample, for 55.17% of pairs the patient had a lower PPI value than the directly matched HC, with a mean (±*SD*) pairwise difference (HC–patient) in PPI of 4.64% (±13.45). That is, particularly in the stroke and PS samples the majority of patients showed lower PPI values in direct comparison with their respective HC matched for age, sex and education level.

#### ANOVAs

For each clinical sample, an ANOVA was computed with *group* (patients vs. HC) as a between‐subjects factor and *age*, *sex* and *education level* as covariates. Separate ANOVAs were computed for PPI, ITT and MET as dependent variables, respectively. Variables that were not normally distributed were Box–Cox transformed before entering the ANOVA models (see Supporting Information, Subsection 2.1). Here, the main effect of *group* for each sample and each outcome variable is reported. For a complete overview of test statistics, please see Supporting Information, Subsection 2.2.

**FIGURE 3:**
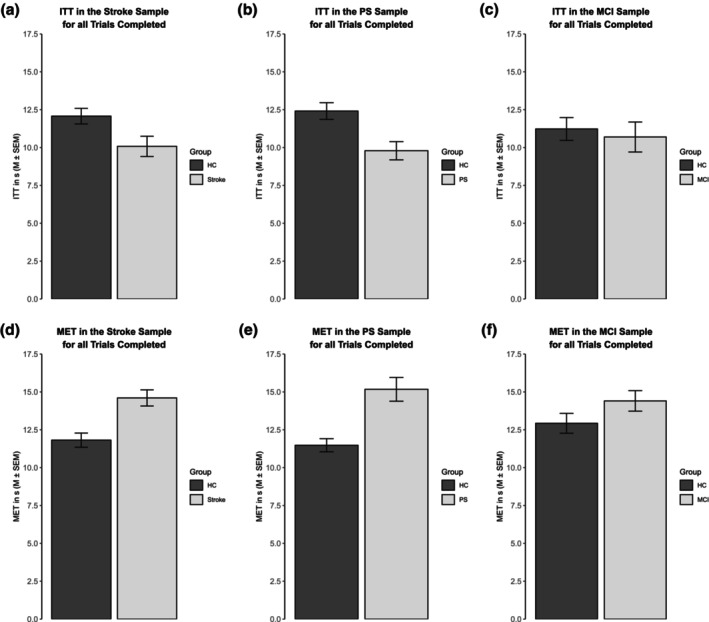
Initial thinking times (ITT) and movement execution times (MET) in clinical samples. Mean initial thinking times (ITT; panels a to c) and movement execution times (MET; panels d to f) in clinical samples. Error bars denote the standard error of the mean (SEM). MCI, mild cognitive impairment; PS, Parkinson syndrome.

In the stroke sample, the ANOVA on PPI yielded a significant effect of *group* (*F*_(1,112)_ = 25.698, *p* < .001) of medium effect size ($\eta_{G}^{2}$ = .187), with stroke patients having significantly lower PPI values than HC (Figure 2a; Table 3). The ANOVA on ITT also yielded a significant *group* effect (*F*_(1,112)_ = 7.732, *p* = .006; $\eta_{G}^{2}$ = .065), with stroke patients having significantly shorter ITT than HC (see Figure 3, panel a). Likewise, the ANOVA on MET revealed a significant *group* effect (*F*_(1,112)_ = 21.023, *p* < .001; $\eta_{G}^{2}$ = .158), with patients having longer MET than HC (see Figure 3, panel d).

In the PS sample, there was a significant effect of *group* on PPI values (*F*_(1,94)_ = 19.412, *p* < .001) of medium effect size ($\eta_{G}^{2}$ = .171), with PS patients having lower PPI values than HC (Figure 2b). The ANOVA on ITT also yielded a significant *group* effect (*F*_(1,94)_ = 8.639, *p* = .004; $\eta_{G}^{2}$ = .084), with PS patients having significantly shorter ITT (Figure 3b). In the ANOVA on MET, *group* also had a significant effect (*F*_(1,94)_ = 16.267, *p* < .001; $\eta_{G}^{2}$ = .148), with PS patients having longer MET than HC (Figure 3e).

In the MCI sample, the ANOVA on PPI did not yield significant differences between MCI patients and HC (*group*: *F*_(1,50)_ = 2.483, *p* = .121, $\eta_{G}^{2}$ = .047). Similarly, neither the ANOVA on ITT nor on MET revealed significant *group* effects (ITT: *F*_(1,50)_ = 0.131, *p* = .719, $\eta_{G}^{2}$ = .003; MET: *F*_(1,50)_ = 2.104, *p* = .153, $\eta_{G}^{2}$ = .040; see Figure 3c,f).

In sum, in the stroke and PS samples, there were significant differences in PPI values between patients and their matched healthy controls, with patients having *lower* PPI (Figure 2). These differences were paralleled by significantly *shorter* ITT and significantly *longer* MET in both patient groups compared to their respective HC group (Figure 3). In both samples, the differences between patients and controls in PPI and MET were of medium effect size (with PPI yielding slightly higher effect sizes than MET), whereas differences in ITT corresponded to a small effect. By contrast, in the MCI sample patients and healthy controls did not differ in any of the latency variables. Of note, PPI values of MCI patients were comparable to those of the other patient groups, but HC in the MCI sample had lower PPI values than the HC in the other two samples (see Table 3). Where covariates were significant, younger age, higher education, and male sex were associated with higher values for PPI and ITT and lower values for MET across all samples (see Supporting Information, Subsection 2.2).

#### Control analyses for correct trials

Given that significant differences in accuracy between patients and HC groups have been shown for all three samples (Köstering, Schmidt, et al., 2015; also see Table 3) and given that accuracy correlated positively with PPI in all samples (see below), group differences in PPI could potentially be confounded by lower accuracy in patient groups. Therefore, we repeated the sample‐wise ANOVAs based on correct trials only. In all three samples, effects closely mirrored those in the main analyses, that is, significant group differences in PPI, ITT and MET in the Stroke and PS samples and no group differences in the MCI sample (see Supporting Information, Subsection 2.3 for details).

#### Control analyses for matched trials

Furthermore, it was tested whether the pattern of group differences pertained for trials matched between each patient–HC pair. That is, significant group differences in the latency variables could be potentially confounded by patients completing fewer trials due to their reduced overall accuracy. Therefore, in these control analyses only trials completed both by a given patient and their matched HC were analysed. For instance, if an HC completed trial #11, but the patient did not complete trial #11 due to the time out, trial #11 was discarded for this patient–HC pair. As this resulted in a reduced number of trials overall, only those patient–HC pairs entered analyses that had at least eight matched trials completed (stroke: 57 pairs; PS: 49 pairs; MCI: 26 pairs). For these matched‐trial ANOVAs, the pattern of group differences remained the same, with significant differences in PPI, ITT and MET in the Stroke and PS samples and no significant group differences in the MCI sample (see Supporting Information, Subsection 2.4 for details).

#### Correlations

Due to the non‐normal distribution of most ITT and MET variables (see Supporting Information), Kendall's tau‐b instead of Pearson correlations was computed throughout. Given that non‐parametric correlations usually yield lower estimates than parametric correlations (El‐Hashash & Shiekh, 2022), this represents a conservative estimate of the association between accuracy and the normally distributed PPI.

In all three clinical samples, there was a significant positive correlation between accuracy and PPI (stroke sample: *τ* = .494, 95% CI [0.345, 0.618], *p* < .001; PS sample: *τ* = .428, 95% CI [0.151, 0.473], *p* < .001; MCI sample: *τ* = .290, 95% CI [0.035, 0.510], *p* = .002), corresponding to a medium effect size in the stroke and PS samples and a small effect size in the MCI sample. Accuracy correlated significantly with ITT in the stroke and PS samples (stroke sample: *τ* = .321, 95% CI [0.277, 0.570], *p* < .001; PS sample: *τ* = .241, 95% CI [0.049, 0.416], *p* = .001), but not in the MCI sample (*τ* = .120, 95% CI [−0.143, 0.367], *p* = .198). This corresponded to a medium effect size in the stroke sample and a small effect size in the PS sample. Accuracy further correlated significantly in a negative direction with MET in all samples (stroke sample: *τ* = −.463, 95% CI [−0.593, −0.310], *p* < .001; PS sample: *τ* = −.478, 95% CI [−0.615, −0.313], *p* < .001; MCI sample: *τ* = −.295, 95% CI [−0.515, −0.040], *p* = .002), corresponding to medium effect sizes in the stroke and PS samples and a small effect in the MCI sample. In sum, associations of accuracy with PPI and MET were consistently found in all clinical samples and were of comparable medium effect size, whereas the association of accuracy with ITT was smaller in effect size and only found in the PS and stroke samples.

Supplementary group‐wise correlations (see Subsection 2.5, Supporting Information) showed that the positive correlation between accuracy and PPI was also significant in all patient and HC groups except for the HC group in the MCI sample (*τ* = .215, *p* = .119).

## DISCUSSION

The current study investigated the test–retest reliability and the known‐group validity of the newly proposed pre‐planning index (PPI) on the Tower of London (TOL) planning task. As regards the first aim, test–retest reliability in a large sample of young healthy participants was mainly measured by the intraclass correlation coefficient ICC(A,1) for absolute agreement, which takes the absolute variation in individual scores, rather than only the rank order of scores, into account (McGraw & Wong, 1996). PPI exhibited the highest absolute agreement with an ICC(A,1) of .726, indicating adequate reliability (cf. Strauss et al., 2006). By contrast, initial thinking time (ITT) yielded moderate reliability with an ICC(A,1) of .666 and movement execution time (MET) showed only low reliability with an ICC(A,1) of .449. Likewise, the session effect in the repeated‐measures ANOVAs, albeit significant for all three variables, showed the smallest effect size for PPI (generalised *η* ^2^ [$\eta_{G}^{2}$] = .027) compared to ITT and MET ($\eta_{G}^{2}$ = .048 and .197, respectively), with PPI having a mean difference between sessions of less than 1% (see Figure 1). The absolute variability in individual scores over repeated testing was further gauged by the coefficient of variation (CV) and the minimal difference (MD) that is needed to reflect a true change in scores with 95% confidence (see Methods for details). PPI showed the lowest variability with a CV of 8.83%, whereas the CV for ITT and MET was considerably higher at 17.16% and 16.33%, respectively. For PPI, the MD was 13.13%, that is, a difference of 13% or more in PPI scores can be considered a true change. This amounts to approximately 21% of the grand mean over testing sessions. By comparison, the MD for ITT and MET amounted to 48% and 49% of their grand mean, respectively. That is, the difference needed to infer a true change in scores beyond possible measurement error – for instance when evaluating patients before and after a treatment – is much higher for the separate latency measures than for PPI.

In sum, as hypothesized, consistently over all indices PPI showed higher reliability and lower individual variability over repeated testing than ITT and MET, with indices for PPI indicating adequate reliability. Hence, PPI seems to be much less prone to task familiarity and/or practice effects, which typically unfold in reduced latencies on repeated testing (Köstering, Nitschke, et al., 2015; Tyburski et al., 2021), than ITT and MET. It has been proposed that the time spent on pre‐planning a solution on the TOL and other planning tasks could be influenced not only by the difficulty of the task itself and by specific instructions (Phillips et al., 2001; Unterrainer et al., 2003), but also by personal processing style, that is, that problem solvers differ in whether they generally exhibit a low or high level of pre‐planning (Davies, 2003, 2005). Of course, such a personal propensity for pre‐planning can also change over time, for example, when problem solvers become more experienced with a given task and develop specific strategies (Davies, 2003). Notwithstanding this, PPI likely taps into this intra‐individually relatively stable problem‐solving characteristic that is not captured when assessing ITT and MET separately, which is reflected in the higher test–retest reliability of the pre‐planning ratio.

It has to be noted that the current results are different from the results by Köstering, Nitschke, et al. (2015) which also used the TOL–F. In that previous study with participants in a similar age range, absolute agreement for ITT and MET did not exceed 0.350 (Köstering, Nitschke, et al., 2015). By contrast, for the current sample, absolute agreement for ITT and MET was higher with indices at least above 0.4. When comparing the crystallized intelligence of participants between studies, which was measured by the same vocabulary test (Lehrl, 2005), the sample of Köstering, Nitschke, et al. (2015) had a mean IQ of 112, whereas the IQ in the current sample was about 95. That is, putatively owing to the larger sample size, the current sample seems to be more representative than the sample of high‐achieving participants from Köstering, Nitschke, et al. (2015), arguing for using adequate sample sizes in reliability studies (Calamia et al., 2013; EFPA, 2013).

As regards the second aim of the study, known‐group validity of the PPI was investigated by testing for significant differences between neurological patients with known planning deficits and healthy controls (HC). For the stroke and Parkinson's syndromes (PS) samples, patients had significantly lower PPI values than the group of matched HC (Figure 2a,b). Lower pre‐planning was also found for the majority of stroke and PS patients when directly comparing them to their respective matched HC (Figure 2d,e). Importantly, lower PPI values in stroke and PS patients were not only driven by longer execution times (Figure 3d,e), which could be biased by motor deficits in these patient groups, but patients also exhibited significantly shorter initial thinking times (Figure 3a,b) which are devoid of any motor movement. Results thus show that stroke and PS patients devote significantly less of their time on pre‐planning a solution than carefully matched healthy participants. Of note, the effect sizes for PPI ($\eta_{G}^{2}$ = .187 and .171 in the stroke and PS samples, respectively) were considerably higher than for ITT ($\eta_{G}^{2}$ = .065 and .084, respectively) and also higher than for MET ($\eta_{G}^{2}$ = .158 and .148, respectively), further attesting to the validity of PPI for clinical comparisons (cf. Sullivan et al., 2009). Differences in PPI for the Stroke and PS samples also persisted when analysing correct trials only and when analysing only those trials that both patient and HC of a given matched pair had completed (see Supporting Information for details), thus precluding that results were biased by differences between patients and controls in accuracy or in the exact trials completed. For these control analyses, again PPI revealed the highest effect sizes. By contrast, none of the latency measures yielded significant differences between the patients with mild cognitive impairment (MCI) and their matched controls. Whereas significant differences in planning accuracy were found for all three clinical groups investigated here (Köstering, Schmidt, et al., 2015), PPI seems to reveal more specific deficits that cannot be uniformly found in patients. Interestingly, PPI values of MCI patients were comparable to those of stroke and PS patients, but the HC group in the MCI sample had slightly lower PPI values than HC groups in the other samples (Figure 2). As HC were matched on age with their respective patients, the HC group in the MCI sample was older than the two other HC groups (see Methods). Thus, there might be a negative age effect on PPI making it more difficult to find differences between patients and healthy controls in the older age range. This awaits further investigation of course in healthy adults of varying age. Furthermore, despite a negative age effect on accuracy, differences in accuracy between MCI patients and healthy controls emerged nonetheless (Köstering, Schmidt, et al., 2015), so that a possible age effect on PPI cannot be the sole explanation for the lack of group differences in PPI for the MCI sample. Alternatively, MCI sub‐types could be differentially affected, masking overall group effects. On a descriptive level, non‐amnestic MCI in the current sample had slightly higher PPI values (*n* = 5; mean ± standard deviation PPI = 45.53% ±16.20) than amnestic patients (*n* = 23; PPI = 42.16% ±11.51) and single‐domain MCI patients had slightly higher PPI values (*n* = 8; PPI = 45.78% ±9.95) than multi‐domain patients (*n* = 20; PPI = 41.55% ±13.01). Future studies with sufficient statistical power for sub‐group analyses could assess whether different MCI sub‐types are indeed differentially affected in their ability for pre‐planning. In sum, significant decrements in pre‐planning were found for both stroke and PS patients that were of greater effect size than effects on planning times and also slightly greater than effects on execution times. When considering potential latency outcome variables for comparing patients' planning performance to that of healthy controls, PPI rather than planning times or execution times should be considered in future studies.

As a third aim, the association of PPI and accuracy was probed, which are conceptualized to be positively related for tasks tapping into meta‐cognitive planning skills (Li et al., 2015). As hypothesized, across both the reliability sample and all clinical samples, there was a significant positive correlation between PPI and accuracy. For the reliability sample, the difference in accuracy correlated positively with the difference in PPI, that is, participants with an increase in accuracy over repeated testing also relatively increased the amount of pre‐planning (or showed less decrease in pre‐planning on repeated testing). The positive relationship between accuracy and PPI was also found for the separate testing sessions in the reliability sample and for all patient and HC groups (except the HC group in the MCI sample) in the clinical samples. That is, there was a consistent positive relationship between pre‐planning and accuracy. Although ITT and MET were also significantly associated with accuracy, for ITT these relationships were smaller in size and not as consistently found as for PPI (for instance, ITT and accuracy were not significantly correlated in the MCI sample). That is, similar to evidence from the Sokoban task (Li et al., 2015, 2020), for the TOL the association of accuracy with a pre‐planning index is greater than with ITT, thereby underlining the conceptual validity of PPI. For MET, the negative correlations with accuracy mostly exceeded those of PPI, but given that MET exhibited markedly lower reliability than PPI, the latter can be regarded as the latency variable that is the most reliable and most consistently associated with accuracy.

Current results have to be interpreted in light of several limitations. First, test–retest reliability of PPI was tested only in young healthy adults, thus limiting the generalizability of these reliability indices to wider age ranges in the general population and to clinical samples. The current results can thus be regarded as a first step in probing the test–retest reliability of the novel PPI that future studies should expand on by testing healthy participants across the whole life span and/or clinical populations. Furthermore, a relatively short retest interval of 1 week was chosen here, which should be complemented in future studies by longer retest intervals and possibly also by including a third testing session. This would allow for better delineation of random measurement error from systematic practice and/or familiarity effects which – for accuracy – have been found to be strongest from first to second session and attenuate from second to a third testing session (Lemay et al., 2004). With regard to measuring PPI in clinical groups, present results were robust across several control analyses and were driven not only by longer execution times, but also shorter planning times in stroke and PS patients. Notwithstanding this, the contribution of reduced motor speed versus increased online planning to increased MET in patients cannot be explicitly delineated from each other. However, it has been shown previously for the PS and stroke patients studied here that they commit significantly more online planning errors and rule breaks than their matched controls (Köstering et al., 2016), which further suggests that longer MET in these patients is significantly driven by insufficient pre‐planning and not only by slower motor speed. Lastly, not all trials administered to participants and patients could be included in the analyses due to the time limit of one minute. This time limit was incorporated following the original version (Shallice, 1982) so as to make the TOL–F amenable to clinical settings where testing time and patients' sustained attention capacities are limited (Kaller, Unterrainer, Kaiser, et al., 2012). However, the TOL–F also allows administration with a three‐minute time limit. Although there is no normative data for this administration mode, it would be useful for research purposes so as to further investigate whether the same pattern of PPI effects can be found when more completed trials can enter statistical analyses. Furthermore, although all participants were thoroughly familiarized with the touch‐screen mode beforehand, which is also more intuitive than handling a computer mouse, a longer time limit could be particularly helpful for participants who do not frequently use computer technology.

## CONCLUSION

In the current study, a novel index to measure pre‐planning on the Tower of London task was proposed and its test–retest reliability and known‐group validity were investigated. Given the widespread use of latency outcome variables for the TOL and related disc‐transfer tasks, the novel PPI represents an alternative to evaluating planning and movement times separately that features a higher test–retest reliability and higher known‐group validity in terms of greater effect sizes in clinical comparisons. However, differences in PPI between patients and healthy controls were not uniformly found in all patient groups, whereas reduced accuracy has been previously found for all patient groups studied here. Planning accuracy can thus be regarded as remaining the primary outcome measure on the TOL and PPI represents a reliable complementary outcome measure that can yield valuable additional information. For instance, patients who impulsively start execution before sufficiently planning a solution and therefore show reduced planning accuracy and reduced pre‐planning might benefit from cognitive training strategies to enhance meta‐cognitive skills, which in turn should positively impact planning accuracy. By contrast, patients with reduced accuracy but preserved or even prolonged pre‐planning ratios might rather benefit from cognitive strategies that directly target the look‐ahead mechanism of planning itself, for instance by practising to break down complex tasks into achievable sub‐goals and to mentally evaluate which actions bring oneself closer to the overall goal. In conclusion, complementing solution accuracy with the novel pre‐planning ratio as an outcome variable on the TOL and related disc‐transfer tasks can enhance both the reliability and validity of how planning ability is measured in health and disease.

## AUTHOR CONTRIBUTIONS

**Lena V. Schumacher:** Conceptualization; writing – original draft; formal analysis; investigation. **Benjamin Rahm:** Formal analysis; writing – review and editing. **Christoph P. Kaller:** Conceptualization; writing – review and editing. **Valentin Schyle:** Investigation; writing – review and editing. **Cornelius Weiller:** Resources; writing – review and editing. **Josef M. Unterrainer:** Resources; conceptualization; writing – review and editing.

## CONFLICT OF INTEREST STATEMENT

CPK and JMU declare to receive a small proportion of the licence fees for the Freiburg version of the Tower of London (TOL‐F) task from the SCHUHFRIED GmbH due to authorship of the published test materials (Kaller, Unterrainer, Kaiser, et al., 2012).

## Supporting information


Appendix S1.

## Data Availability

The data are not publicly available due to privacy or ethical restrictions.

## Footnotes

^1^
For the healthy controls to be matched with the patients the higher cut‐off of 19 points (instead of the usual cut‐off of 13 points) was applied to enable a fair comparison with patient groups who had slightly elevated depressivity scores. For further details, see Köstering, Schmidt, et al. (2015).

^2^
Please note that the numbers for female and male participants given in Table 1 in the publication by Köstering, Schmidt, et al. (2015) are accidentally interchanged in the PS and stroke patient groups.
