# Supplementary material for: A novel index to measure pre‐planning in the Tower of London task: Test–retest reliability and known‐group validity
Source: Br J Psychol. 2025 Nov 28;117(3):1007–25. doi: 10.1111/bjop.70044 (PMC13353248; doi:10.1111/bjop.70044)
Supplement: Supplementary file 1 — Appendix S1. [file BJOP-117-1007-s001.docx]

Supplementary Materials

**A Novel Index to Measure Pre-Planning in the Tower of London Task: Test-Retest Reliability and Known-Group Validity**

Lena V. Schumacher, Benjamin Rahm, Christoph P. Kaller, Valentin Schyle, Cornelius Weiller, and Josef M. Unterrainer

1. Reliability Analyses

## Descriptive Statistics for Correct Trials

**Table S1**

*Descriptive Statistics for Correct Trials in the Reliability Sample*

|  | Session 1 | | |  | Session 2 | | |  | Difference score  (Session 2–Session 1) | | |
| --- | --- | --- | --- | --- | --- | --- | --- | --- | --- | --- | --- |
|  | M | SD | Min–Max |  | M | SD | Min–Max |  | M | SD | Min–Max |
| PPI  (%) | 63.90 | 8.84 | 31.95–82.61 |  | 63.72 | 8.72 | 35.27–79.33 |  | -0.18 | 6.17 | -19.33–21.54 |
| ITT  (s) | 13.33 | 4.53 | 2.83–29.09 |  | 11.30 | 4.09 | 2.62–26.02 |  | -2.03 | 3.21 | -10.11–7.18 |
| MET  (s) | 6.15 | 1.73 | 3.29–16.01 |  | 5.15 | 1.22 | 2.81–9.88 |  | -1.01 | 1.44 | -6.74–5.13 |

*Note*. PPI, pre-planning index; ITT, initial thinking time; MET, movement execution time; ACC, accuracy.

**Figure S1**


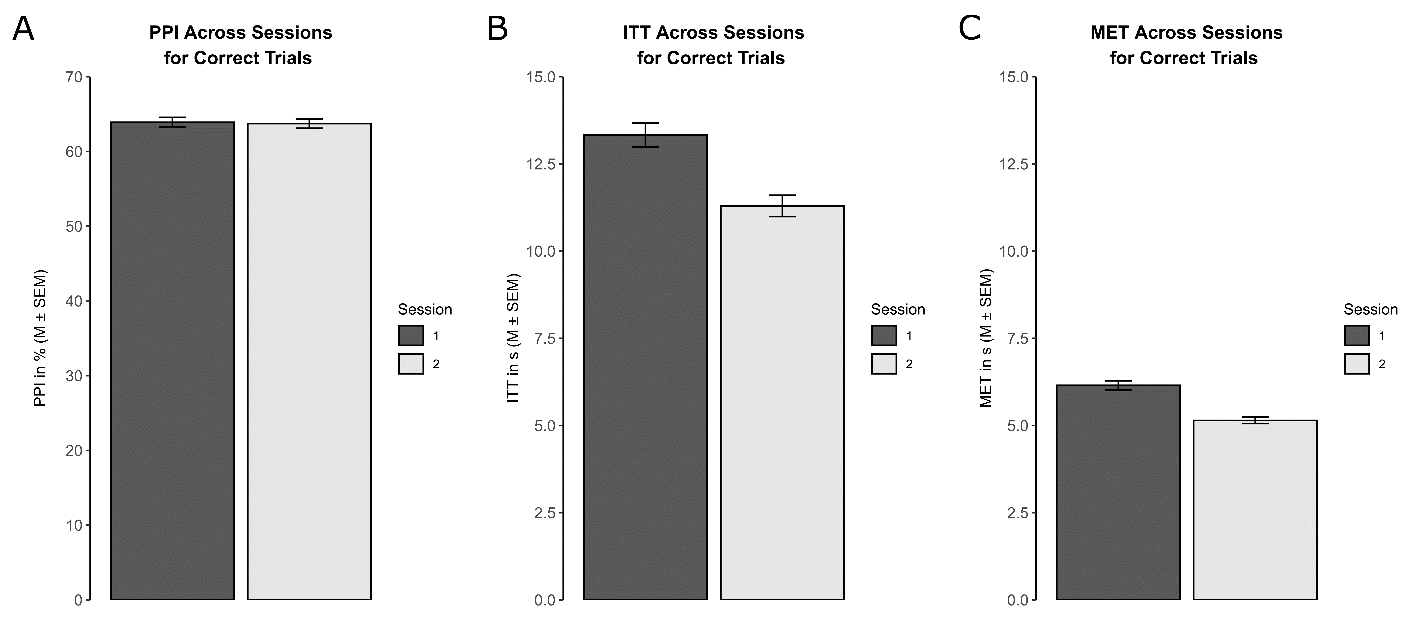
*Planning Latency Variables for Correct Trials Across Sessions*

Note.

## Session-Wise Correlations

*Note*. Mean (A) pre-planning index (PPI), (B) initial thinking times (ITT), and (C) movement execution times (MET) for correct trials only across repeated measurements (dark grey bars, Session 1; light grey bars, Session 2). Error bars denote the standard error of the mean (SEM).

## Quantile-Quantile Plots of Latency Variables and Transformations of Variables

Box-Cox power transformations are commonly applied to positively skewed data (Box & Cox, 1964; Sakia, 1992), but did not yield normally distributed data for the negatively skewed PPI variables of session 1 and 2 (see Figure S2, panels A and B), so they were square-root transformed with the formula √(maximum(PPI+1)-PPI). MET variables for session 1 and session 2 were positively skewed (Fig. S2-E and -F) and Box-Cox transformed with a lamda (λ) of -0.0202 and -0.0606, respectively, using the function *boxcox* of the R package MASS (Venables & Ripley, 2002).

**Figure S2**

*Quantile-Quantile Plots of Latency Variables*


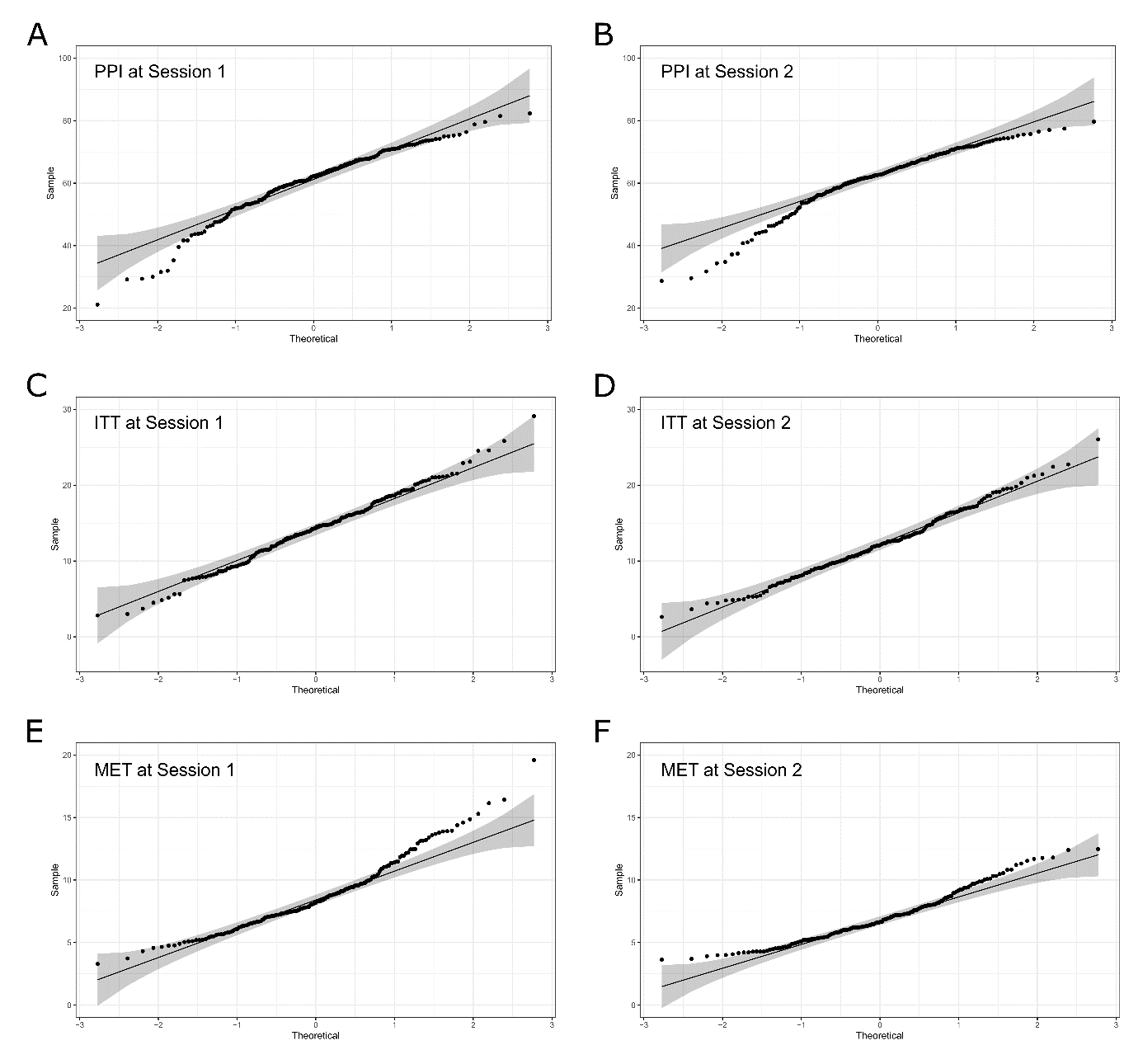


## Session-Wise Correlations

**Table S2**

*Session-Wise Kendall’s tau-b Correlations Between Accuracy and Latency Measures*

|  |  | Session 1 | |  | Session 2 | |
| --- | --- | --- | --- | --- | --- | --- |
|  |  | τ | 95% CI |  | τ | 95% CI |
| ACC–PPI | | .360*** | .225; .481 |  | .345*** | .209; .468 |
| ACC–ITT | | .219*** | -.074; .354 |  | .197*** | .052; .334 |
| ACC–MET | | -.434*** | -.546; -.307 |  | -.456*** | -.565; -.332 |
| PPI–ITT | | .623*** | .524; .705 |  | .624*** | .525; .706 |
| PPI–MET | | -.550*** | -.645; -.439 |  | -.473*** | -.579; -.350 |
| ITT–MET | | -.208*** | -.344; -.064 |  | -.146** | -.286; .001 |

Note. 95% CI, 95% confidence interval; ACC, accuracy; PPI, pre-planning index; ITT, initial thinking time; MET, movement execution time.

*** significant at p < .001, ** significant at p < .01

1. Known-Group Validity Analyses

## Normality Tests of Latency Variables and Transformations of Variables

In the stroke sample, Shapiro-Wilk tests revealed significant deviation from normality for ITT (W = .971, p = .0097) and MET (W = .977, p = .0384), but not for PPI (W = .984, p = .152). ITT and MET were Box-Cox transformed with a lambda (λ) of .3838 for both ITT and MET using the function *boxcox* of the R package MASS (Venables & Ripley, 2002).

In the PS sample, MET deviated significantly from normality (W = .932, p < .001) and was Box-Cox transformed with λ = -.0606. Shapiro-Wilk tests for ITT and PPI both yielded a W of .978 and p-values of .0808 and .0815, respectively. Due to the borderline significance, QQ plots for ITT and PPI were closely inspected, but did also not reveal a significant deviation from normality (data not shown), so that both variables were left untransformed.

In the MCI sample, Shapiro-Wilk tests revealed a significant deviation from normality for ITT (W = .949, p = .0164) and MET (W = .936, p = .0042), but not for PPI (W = .979, p = .410). ITT was Box-Cox transformed with λ = .1414 and MET with λ = -.3030.

## Statistical Effects in Main ANOVA Models

Stroke Sample

| Dependent Variable | Factor | Sum of Squares | df | F | p | η^2^_G_ |
| --- | --- | --- | --- | --- | --- | --- |
| PPI | Group | 2584.293 | 1 | 25.698 | 1.59 х 10^-6^ | .187 |
|  | Sex | 4.693 | 1 | 0.047 | .829 | <.001 |
|  | Age | 2554.645 | 1 | 25.403 | 1.80 х 10^-6^ | .185 |
|  | EdLev | 1572.900 | 4 | 3.910 | .005 | .123 |
|  | Residuals | 11263.19 | 112 |  |  |  |
| ITT | Group | 7.805 | 1 | 7.732 | .006 | .065 |
|  | Sex | 0.113 | 1 | 0.112 | .738 | .001 |
|  | Age | 4.529 | 1 | 4.486 | .036 | .039 |
|  | EdLev | 7.660 | 4 | 1.897 | .116 | .063 |
|  | Residuals | 113.058 | 112 |  |  |  |
| MET | Group | 9.298 | 1 | 21.023 | 1.19 х 10^-5^ | .158 |
|  | Sex | 0.261 | 1 | 0.590 | .444 | .005 |
|  | Age | 16.257 | 1 | 36.756 | 1.85 х 10^-8^ | .247 |
|  | EdLev | 4.463 | 4 | 2.523 | .045 | .083 |
|  | Residuals | 49.536 | 112 |  |  |  |

*Note*. PPI, pre-planning index; ITT, initial thinking time; MET, movement execution time; EdLev, Education Level, η^2^_G_, generalised eta squared.

PS Sample

| Dependent Variable | Factor | Sum of Squares | df | F | p | η^2^_G_ |
| --- | --- | --- | --- | --- | --- | --- |
| PPI | Group | 2468.863 | 1 | 19.412 | 2.79 х 10^-5^ | .171 |
|  | Sex | 31.448 | 1 | 0.247 | .620 | .003 |
|  | Age | 1043.566 | 1 | 8.205 | .005 | .080 |
|  | EdLev | 2017.133 | 4 | 3.965 | .005 | .144 |
|  | Residuals | 11955.21 | 94 |  |  |  |
| ITT | Group | 138.618 | 1 | 8.639 | .004 | .084 |
|  | Sex | 0.026 | 1 | 0.002 | .968 | <.001 |
|  | Age | 24.415 | 1 | 1.522 | .220 | .016 |
|  | EdLev | 163.134 | 4 | 2.542 | .045 | .098 |
|  | Residuals | 1508.321 | 94 |  |  |  |
| MET | Group | 0.932 | 1 | 16.267 | 1.12 х 10^-4^ | .148 |
|  | Sex | 0.0002 | 1 | 0.004 | .953 | <.001 |
|  | Age | 0.788 | 1 | 13.742 | 3.54 х 10^-4^ | .128 |
|  | EdLev | 1.431 | 4 | 6.243 | 1.69 х 10^-4^ | .210 |
|  | Residuals | 5.387 | 94 |  |  |  |

*Note*. PPI, pre-planning index; ITT, initial thinking time; MET, movement execution time; EdLev, Education Level, η^2^_G_, generalised eta squared.

MCI Sample

| Dependent Variable | Factor | Sum of Squares | df | F | p | η^2^_G_ |
| --- | --- | --- | --- | --- | --- | --- |
| PPI | Group | 225.337 | 1 | 2.483 | .121 | .047 |
|  | Sex | 424.245 | 1 | 4.675 | .035 | .086 |
|  | Age | 67.622 | 1 | 0.745 | .392 | .015 |
|  | EdLev | 954.876 | 4 | 2.630 | .045 | .174 |
|  | Residuals | 4537.598 | 50 |  |  |  |
| ITT | Group | 0.162 | 1 | 0.540 | .466 | .011 |
|  | Sex | 0.674 | 1 | 2.247 | .140 | .043 |
|  | Age | 0.005 | 1 | 0.018 | .895 | <.001 |
|  | EdLev | 3.649 | 4 | 3.041 | .025 | .196 |
|  | Residuals | 15 | 50 |  |  |  |
| MET | Group | 0.028 | 1 | 2.104 | .153 | .040 |
|  | Sex | 0.003 | 1 | 0.221 | .640 | .004 |
|  | Age | 0.022 | 1 | 1.697 | .199 | .033 |
|  | EdLev | 0.031 | 4 | 0.597 | .667 | .046 |
|  | Residuals | 0.658 | 50 |  |  |  |

*Note*. PPI, pre-planning index; ITT, initial thinking time; MET, movement execution time; EdLev, Education Level, η^2^_G_, generalised eta squared.

## 2.3 Control Analyses for Correct Trials Only

For all clinical samples, all ANOVAs were repeated based on *correct trials only* instead of *all trials completed*. As for the main analyses, variables were tested for adherence to normality assumptions with Shapiro-Wilk tests and by inspection of QQ Plots and were Box-Cox transformed if they significantly deviated from normality.

For the Stroke sample, the ANOVA on PPIcorr (PPI for correct trials) yielded a significant effect of *group* (F_(1,112)_ = 28.779, p < .001) of medium effect size (generalised eta-squared [η^2^_G_] = .204) as well as significant effects of the covariates *age* (F_(1,112)_ = 32.409, p < .001, η^2^_G_ = .224) and *education level* (F_(4,112)_ = 3.810, p = .006, η^2^_G_ = .120). For ITTcorr (ITT for correct trials), there was a significant effect of *group* (F_(1,112)_ = 6.578, p = .012) of small effect size (η^2^_G_ = .055); none of the covariates reached significance (*age*: F = 3.777, p = .054; all other F < 2, p > .129). The ANOVA on METcorr (MET for correct trials) yielded a significant effect of *group* (F_(1,112)_ = 15.834, p < .001) of small effect size (η^2^_G_ = .124) as well as a significant effect of *age* (F_(1,112)_ = 38.270, p < .001, η^2^_G_ = .255).

In the PS sample, the ANOVA on PPIcorr yielded a significant effect of *group* (F_(1,94)_ = 15.027, p < .001) of medium effect size (η^2^_G_ = .138) as well as effects of the covariates *age* (F_(1,94)_ = 8.414, p = .005, η^2^_G_ = .082) and *education level* (F_(4,94)_ = 2.557, p = .044, η^2^_G_ = .098). For ITTcorr, there was only a significant effect of *group* (F_(1,94)_ = 6.594, p = .012; η^2^_G_ = .066; covariates: highest F = 1.481, lowest p = .214). For METcorr, the ANOVA yielded a significant *group* effect (F_(1,94)_ = 12.072, p < .001) of small effect size (η^2^_G_ = .114) and a significant effect of *age* (F_(1,94)_ = 14.905, p < .001, η^2^_G_ = .137).

In the MCI sample, the ANOVA on PPIcorr did not yield an effect of *group* (F_(1,50)_ = 1.715, p = .196, η^2^_G_ = .033), only of the covariate *sex* (F_(1,50)_ = 8.342, p = .006, η^2^_G_ = .143). Likewise, neither for ITTcorr nor for METcorr did the *group* factor reach significance (ITTcorr: F_(1,50)_ = 0.177, p = .676, η^2^_G_ = .004; METcorr: F_(1,50)_ = 2.339, p = .132, η^2^_G_ = .045). There were only significant effects for *education level* on ITTcorr (F_(4,50)_ = 2.840, p = .034, η^2^_G_ = .185) and for *sex* on METcorr (F_(1,50)_ = 4.456, p = .040, η^2^_G_ = .082).

As for the main analyses, there were significant group effects on PPI, ITT, and MET in the Stroke and PS sample, with PPI yielding the highest effect sizes. These differences between patients and HC resulted from lower PPI and lower ITT values in patients as well as higher MET values in patients. Where covariates were significant, the effects broadly mirrored those found for the main analyses, i.e. higher PPI and ITT and lower MET values were associated with younger age, higher education, and male sex.

## Control Analyses for Matched Trials

For all clinical samples, all ANOVAs were repeated based on *matching the exact trials completed by each patient–HC pair*. That is, a given trial only entered analyses if both patient and HC had completed it. As this resulted in a reduced number of trials overall, a cut-off of 8 trials was set, i.e. a patient*–*HC pair entered analyses only if they had completed at least 8 matched trials. This resulted in slightly reduced numbers of pairs (Stroke sample: 57 pairs; PS sample: 49 pairs; MCI sample: 26 pairs). Again, variables were tested for adherence to normality with Shapiro-Wilk tests and by inspection of QQ-Plots and were Box-Cox transformed if they significantly deviated from normality (this was the case for most of the ITT and MET variables; PPI did not significantly deviate from normality in any of the samples).

In the stroke sample, the matched-trials ANOVA for PPI yielded a significant effect of *group* (F_(1,106)_ = 22.317, p < .001) of medium effect size (η^2^_G_ = .174) as well as significant effects of age and education level (age: F_(1,106)_ = 20.049, p < .001, η^2^_G_ = .159; education level: F_(4,106)_ = 4.427, p = .002, η^2^_G_ = .143). In the matched-trials ANOVA on ITT, there was a significant *group* effect (F_(1,106)_ = 5.918, p = .017) of small effect size (η^2^_G_ = .053), but no significant effects of the covariates (F = 3.441, p = .066 for age; F = 2.079, p = .089 for education level). The matched-trials ANOVA on MET yielded a significant *group* effect (F_(1,106)_ = 19.702, p < .001) of medium effect size (η^2^_G_ = .157) as well as significant effects of the covariates age (F_(1,106)_ = 28.719, p < .001, η^2^_G_ = .213) and education level (F_(4,106)_ = 3.290, p = .014, η^2^_G_ = .110).

In the PS sample, the matched-trials ANOVA on PPI yielded a significant effect of *group* (F_(1,90)_ = 16.337, p < .001) of medium effect size (η^2^_G_ = .154). The covariates *age* and *education level* also reached significance (age: F_(1,90)_ = 8.447, p = .005, η^2^_G_ = .086; education level: F_(4,90)_ = 4.015, p = .005, η^2^_G_ = .151). In the matched-trials ANOVA on ITT, there was a significant effect of *group* (F_(1,90)_ = 8.690, p = .004) of small effect size (η^2^_G_ = .088) and of *education level* (F_(4,90)_ = 2.725, p = .034, η^2^_G_ = .108). Lastly, the matched-trials ANOVA on MET yielded a significant group effect (F_(1,90)_ = 12.569, p < .001) of small effect size (η^2^_G_ = .123) as well as effects of *age* and *education level* (age: F_(1,90)_ = 12.717, p < .001, η^2^_G_ = .124; education level: F_(4,90)_ = 6.208, p < .001, η^2^_G_ = .216).

In the MCI sample, the matched-trials ANOVA on PPI did not yield an effect of *group* (F_(1,44)_ = 2.964, p = .092, η^2^_G_ = .063) or of any of the covariates (highest F = 2.497, lowest p = .121). Likewise, neither for ITT nor for MET did the *group* factor reach significance (ITT: F_(1,44)_ = 2.290, p = .137, η^2^_G_ = .049; MET: F_(1,44)_ = 0.480, p = .492, η^2^_G_ = .011). Further, none of the covariates reached significance in the two models (*education level* on ITT: F = 2.319, p = .071; all other covariates: highest F = 2.033, lowest p = .161).

As for the main analyses, there were significant group effects on PPI, ITT, and MET in the Stroke and PS sample, with PPI yielding the highest effect sizes. Across models, these group effects resulted from lower PPI, lower ITT, and higher MET values in patients compared to HC. Again, where covariates were significant, higher PPI and ITT and lower MET values were associated with younger age and higher education. Sex did not reach significance in any of the models (highest F = 2.497, lowest p = .121).

## Correlations of Accuracy with Latency Variables

**Table S3**

*Correlations of Accuracy with Latency Variables in Patient and Healthy Control Groups Across Clinical Samples*

|  |  | Stroke | |  | PS | |  | MCI | |
| --- | --- | --- | --- | --- | --- | --- | --- | --- | --- |
|  |  | τ | 95% CI |  | τ | 95% CI |  | τ | 95% CI |
| ACC– PPI | Patients | .525*** | .312; .687 |  | .405*** | .145; .612 |  | .271* | -.106; .580 |
|  | HC | .353*** | .109; .557 |  | .212* | -.068; .461 |  | .215 | -.165; .539 |
| ACC– ITT | Patients | .397*** | .160; .592 |  | .211* | -.069; .460 |  | .074 | -.301; .428 |
|  | HC | .111 | -.147; .355 |  | .034 | -.244; .307 |  | .098 | -.278; .448 |
| ACC– MET | Patients | -.430*** | -.616; -.197 |  | -.538*** | -.709; -.308 |  | -.276* | -.584; .100 |
|  | HC | -.422*** | -.610; -.188 |  | -.310** | -.539; -.038 |  | -.209 | -.535; .170 |

*Note*. PS, Parkinson syndrome; MCI, Mild Cognitive Impairment; 95% CI, 95% confidence interval; ACC, accuracy; PPI, pre-planning index; ITT, initial thinking time; MET, movement execution time.

*** significant at p < .001, ** significant at p < .01, * significant at p < .05

References

Box, G. E. P., & Cox, D. R. (1964). An Analysis of Transformations. *Journal of the Royal Statistical Society. Series B (Methodological)*, *26*(2), 211–252.

Sakia, R. M. (1992). The Box-Cox Transformation Technique: A Review. *The Statistician*, *41*(2), 169. https://doi.org/10.2307/2348250

Venables, W. N., & Ripley, B. D. (2002). *Modern Applied Statistics with S*. Springer. https://www.stats.ox.ac.uk/pub/MASS4/
